# Ecological momentary assessment and cue-elicited drug craving as primary endpoints: study protocol for a randomized, double-blind, placebo-controlled clinical trial testing the efficacy of a GLP-1 receptor agonist in opioid use disorder

**DOI:** 10.1186/s13722-024-00481-7

**Published:** 2024-07-27

**Authors:** Christopher S. Freet, Brianna Evans, Timothy R. Brick, Erin Deneke, Emily J. Wasserman, Sarah M. Ballard, Dean M. Stankoski, Lan Kong, Nazia Raja-Khan, Jennifer E. Nyland, Amy C. Arnold, Venkatesh Basappa Krishnamurthy, Julio Fernandez-Mendoza, H. Harrington Cleveland, Adam D. Scioli, Amanda Molchanow, Amy E. Messner, Hasan Ayaz, Patricia S. Grigson, Scott C. Bunce

**Affiliations:** 1 Department of Psychiatry and Behavioral Health, The Pennsylvania State University College of Medicine, Hershey, PA USA; 2 Department of Neural and Behavioral Sciences, The Pennsylvania State University College of Medicine, Hershey, PA USA; 3 Department of Human Development and Family Studies, The Pennsylvania State University, University Park, PA USA; 4 Institute for Computational and Data Sciences, The Pennsylvania State University, University Park, PA USA; 5 Fran and Doug Tieman Center for Research, Caron Treatment Centers, Wernersville, PA USA; 6 Department of Public Health Sciences, The Pennsylvania State University College of Medicine, Hershey, PA USA; 7 Department of Medicine, The Pennsylvania State University College of Medicine, Hershey, PA USA; 8 Department of Obstetrics & Gynecology, The Pennsylvania State University College of Medicine, Hershey, PA USA; 9 School of Biomedical Engineering, Science and Health Systems, Drexel University, Philadelphia, PA USA; 10 Department of Medicine and Psychiatry, Division of Pulmonary, Allergy, Critical Care and Sleep Medicine, University of Pittsburgh, Pittsburgh, PA USA; 11 Synergy Pharmacy Services LLC, Norristown, PA USA; 12 Penn State University College of Medicine, Milton S. Hershey Medical Center, H073, 500 University Drive, Hershey, PA 17033-0850 USA

**Keywords:** Glucagon-like peptide 1 receptor agonist, GLP-1RA, Craving, Substance use disorder, Liraglutide, Functional near infrared spectroscopy, fNIRS, EMA

## Abstract

**Background:**

Despite continuing advancements in treatments for opioid use disorder (OUD), continued high rates of relapse indicate the need for more effective approaches, including novel pharmacological interventions. Glucagon-like peptide 1 receptor agonists (GLP-1RA) provide a promising avenue as a non-opioid medication for the treatment of OUD. Whereas GLP-1RAs have shown promise as a treatment for alcohol and nicotine use disorders, to date, no controlled clinical trials have been conducted to determine if a GLP-1RA can reduce craving in individuals with OUD. The purpose of the current protocol was to evaluate the potential for a GLP-1RA, liraglutide, to safely and effectively reduce craving in an OUD population in residential treatment.

**Method:**

This preliminary study was a randomized, double-blinded, placebo-controlled clinical trial designed to test the safety and efficacy of the GLP-1RA, liraglutide, in 40 participants in residential treatment for OUD. Along with taking a range of safety measures, efficacy for cue-induced craving was evaluated prior to (Day 1) and following (Day 19) treatment using a Visual Analogue Scale (VAS) in response to a cue reactivity task during functional near-infrared spectroscopy (fNIRS) and for craving. Efficacy of treatment for ambient craving was assessed using Ecological Momentary Assessment (EMA) prior to (Study Day 1), across (Study Days 2–19), and following (Study Days 20–21) residential treatment.

**Discussion:**

This manuscript describes a protocol to collect clinical data on the safety and efficacy of a GLP-1RA, liraglutide, during residential treatment of persons with OUD, laying the groundwork for further evaluation in a larger, outpatient OUD population. Improved understanding of innovative, non-opioid based treatments for OUD will have the potential to inform community-based interventions and health policy, assist physicians and health care professionals in the treatment of persons with OUD, and to support individuals with OUD in their effort to live a healthy life. Trial registration: ClinicalTrials.gov: NCT04199728. Registered 16 December 2019, https://clinicaltrials.gov/study/NCT04199728?term=NCT04199728.

**Protocol Version:**

10 May 2023

## Background

Approximately 2.7 million people in the United States reported suffering from opioid use disorder (OUD) in 2020 [1]. Research indicates that the rate of opioid overdoses increased consistently over the last two decades (9,496 in 2001 to 80,411 in 2021; 2). Studies indicate that psychosocial interventions alone provide relatively poor outcomes in OUD recovery [2, 3]. Pharmacological treatments for OUD, however, continue to be limited to three medications. These medications for opioid use disorder (MOUD), i.e.,methadone, buprenorphine, and naltrexone, do provide effective treatment pathways for recovery maintenance compared with no treatment or behavioral therapies alone [4, 5], but these treatment avenues still suffer from high attrition and relapse rates [2, 6]. Treatment retention rates for methadone, on average, drop from 84% at 24 weeks [7] to between 54 − 35% at 1 year [8, 9]. Similarly, average retention rates using buprenorphine drop from 59% at 24 weeks [7] to 23% at 1 year [8]. Moreover, these MOUDs are synthetic opioids, which give rise to their own limitations and drawbacks. As full and partial opioid agonists, respectively, methadone and buprenorphine can carry the risk of misuse, respiratory depression, overdose during induction and stabilization, or overdose when taken with other opioids or medications, and are underused [2, 6, 10–13]. Because these medications are full or partial opioid agonists, many people will not use currently available MOUD due to familial or work-related stigma. Many health care providers refuse to prescribe MOUD, and several professions do not allow the use of full or partial opioid agonists while on the job [14–17]. Naltrexone, a µ-opioid antagonist, requires abstinence before initiating use, suffers from low rates of adherence, and lacks tolerability [18–20]. As the global opioid epidemic continues, novel, safe, effective, and tolerable medications need to be developed and approved.

A current, promising avenue of interest is in the repurposing of glucagon-like peptide 1 receptor agonist (GLP-1RA) medications to treat OUD, specifically to address the craving inherent in OUD and to improve abstinence and recovery rates. Peripherally, GLP-1 is a hormone produced by L cells in the small intestine that reduces intake, in part, by increasing insulin release, decreasing glucagon release, and decreasing gastric emptying [21]. Centrally, GLP-1 is an endogenous neuropeptide produced in the caudal portion of the nucleus of the solitary tract (NST). Efferent projections terminate in a wide range of dorsomedial and paraventricular hypothalamic nuclei, as well as mesolimbic nuclei such as the ventral tegmental area and the nucleus accumbens [22, 23]. Behaviorally, GLP-1 is in a position to regulate feeding behaviors in general [24, 25] and, more specifically, to influence satiety, perception of hedonic value, and even motivation. In support, infusion of GLP-1 into the paraventricular nucleus (PVN) decreases intake of a liquid diet [26], as well as feeding behaviour [27], in rats. Additionally, GLP-1 analogue activation in mesolimbic nuclei also attenuates food intake, and this finding has been linked to a decrease in the perception of food reward/reinforcement as rats administered the GLP-1 receptor agonist, Exendin-4, exhibit reduced conditioned place preference for chocolate pellets and reduced progressive-ratio responding for sucrose and sweetened fat [28, 29]. Findings of this nature, and those also showing improved glucose regulation [30–32], have led to FDA approval for the use of GLP-1RAs for the treatment of type 2 diabetes mellitus and obesity in humans. That said, the safety and efficacy of GLP-1RAs for the treatment of SUD has yet to be comprehensively evaluated.

There is a growing body of literature demonstrating that GLP-1RAs may decrease intake and motivation not only for food, but for opioids as well, suggesting that these agonists may be an effective treatment in OUD. Preclinically, GLP-1RAs decrease drug self-administration [33–35] and drug seeking/reinstatement behaviour [36–39] for heroin, oxycodone, and fentanyl in rats. Current clinical investigations have also begun to evaluate these effects with other drug use such as alcohol, nicotine, and cocaine, in persons with SUD. A double-blind, placebo-controlled clinical trial using exenatide in patients with alcohol dependence used fMRI to demonstrate attenuated cue reactivity to alcohol in the ventral striatum and septal area. However, heavy drinking days and total alcohol intake were not significantly different compared with placebo, with the exception of a subgroup with class I obesity or greater [40, 41]. Currently, as per ClinicalTrials.gov, two other clinical trials in Denmark are studying the effects of GLP-1R agonists on alcohol use disorder (AUD; NCT03232112 and NCT05895643), with results pending. Additionally, trials specifically evaluating semaglutide in AUD (NCT05891587, NCT05892432, NCT05520775, NCT06015893, NCT05895643) are currently underway. One double-blind, placebo-controlled, randomized clinical trial has been published demonstrating that exenatide, combined with nicotine replacement therapy, attenuated craving and withdrawal, and increased smoking abstinence, in prediabetic and/or overweight treatment-seeking smokers [42, 43]. Ongoing clinical trials are currently evaluating the effect of GLP-1RAs on nicotine (NCT03712098, NCT03204396, and NCT05530577), as well as concurrent alcohol and nicotine use (NCT02690987). Finally, with respect to cocaine, a double-blind, within-subject design using acute exenatide pre-treatment in patients with cocaine use disorder did not observe a change in cocaine intake, wanting, or self-reported euphoria. The use of only one dose of exenatide, and a single, acute treatment, limited the ability to draw clear conclusions regarding efficacy from this study [44]. To our knowledge, however, the described study is the first to evaluate the capacity of a GLP-1RA to attenuate craving in humans seeking treatment for OUD.

## The current study

The purpose of the current protocol was to test whether once daily treatment with the GLP-1RA, liraglutide, can safely and effectively reduce craving in patients in a residential treatment facility for OUD while they are receiving counselling with or without the common MOUD, buprenorphine/naloxone (BUP/NA). The described study is a double-blind, randomized trial with treatment (GLP-1RA, liraglutide) and control (placebo) arms. Over the 21-day protocol, participants completed clinical assessments and pre-treatment tasks (Day 1), received study treatment for 18 days (either liraglutide or placebo; Days 2–19), completed post-treatment tasks (Day 19), were monitored for rebound effects from discontinuation of the study treatment (Days 20–21), and contacted to assess Adverse Events (AE) 30 days following Day 19.

Liraglutide has been approved by the FDA for the treatment of Type 2 diabetes mellitus since 2010, and for the treatment of obesity since 2014. Although this medication has been shown to be safe and effective in these populations for over a decade, it has not been well evaluated in an OUD population, and, as such, appropriate safety measures were included in the current proposal. For example, as liraglutide is used to regulate glycemic control in diabetes management and to lower body weight in obese patients, indices of glucose metabolism and daily body weight were evaluated during the study. Because opioid use can suppress respiratory rate, cardiorespiratory measures were taken across the study to evaluate possible interactions with the GLP-1RA.

Craving, a multidimensional phenomenon with physiological, neurochemical, subjective, and behavioral correlates [45], has been central to theories of addiction for more than seven decades, particularly as a predictor of continued use and relapse to substance use, including opioids [46, 47]. We evaluated the subjective aspect of craving in the current study using ecological momentary assessment (EMA) before, across, and following the study treatment. EMA assessments of craving during residential OUD treatment have been shown to have high reliability, sensitivity, and utility, suggesting that EMA is an effective way to measure craving in this population [48]. In addition, we evaluated the cue-induced aspect of craving using a visual analogue scale (VAS) craving questionnaire presented before and after performance in a cue reactivity task using functional near-infrared spectroscopy (fNIRS). Blunted response to natural reward (non-drug) cues, compared with drug cues, in cue reactivity tasks has been demonstrated in individuals with OUD, using functional magnetic resonance imaging (fMRI) [49–52], and this pattern has been associated with future opioid use [53]. Studies employing fNIRS, a translational neuroimaging technology that uses light to measure changes in the blood oxygen level dependent (BOLD) signal, have generally found results similar to the fMRI studies [54–58].

## Methods

### Aims

The aims of the current protocol were: (1) to evaluate the safety of liraglutide, administered daily, in humans seeking treatment for OUD, and (2) to evaluate the efficacy of liraglutide (vs. placebo), administered daily, in reducing craving for opioids in humans in residential treatment for an OUD.

### Outcomes

The primary outcomes for this study were as follows. **(1) To determine whether liraglutide can reduce cue-elicited drug craving over 19 days while participants are in residential treatment for OUD.** Self-reported cue-elicited drug craving from baseline (Day 1) to the end of the target drug dose (Day 19) was measured on a 0–100-point visual analogue scale (VAS), where 0 = no craving and 100 = maximum craving. Assessments were taken prior to, and immediately after, exposure to visual drug cues in the Cue Reactivity Task. **(2) To study whether liraglutide can reduce ambient (daily) drug craving over approximately 21 days while participants are in residential treatment for OUD.** Change in ambient drug craving over time was measured throughout the study (Days 1–21) using a 0-100 VAS delivered via smartphone using (EMA; 60) 4 times per day, on each test day (Days 1 and 19), on the first two days and last 2 days of each treatment dose (Days 2–3, 6–9, 12–15, 18–19), and during the rebound period (Days 20 and 21).

The secondary outcomes of this pilot study were designed to evaluate the safety of daily administration of liraglutide in humans seeking treatment for an OUD. **1) Cardiorespiratory Function**: Blood pressure, heart rate, and respiration changes from baseline (Day 1) to: (i) the first administration of each study drug dose (Days 2, 8, 14); (ii) the end of the target drug dose (Day 19); and (iii) rebound follow-up (Day 21). **2) Body Weight (kg)**: Absolute and percent change in body weight from baseline through the end of rebound follow-up (Daily, from Days 1 to 21). **3) Glycemic Control**: Glycemic control was monitored by fasting blood samples for fructosamine and HbA1c. Fasting blood sample measurements change slowly over time (i.e., across weeks and months) and therefore were evaluated only on Days 2 and 19 via venipuncture. **4) Adverse Events**: The frequency of adverse events (AE) and serious adverse events (SAE) deemed related to treatment throughout the study period were monitored (Days 1–21, and 30 days post-intervention).

Exploratory outcomes included: **(1) Prefrontal Cortical Response to Drug Cues**: Change in blood oxygenation level response to visual opioid drug cues in prefrontal cortex from baseline (Day 1) to end of the target drug dose (Day 19) using fNIRS; **(2) Rebound Ambient Drug Craving**: Change in ambient drug craving as measured by 0-100 VAS delivered via a smartphone using EMA from the end of the target drug dose (Day 19) to rebound follow up (Day 21); **(3) Rebound Blood Pressure**: Change in blood pressure from end of the target drug dose (Day 19) to rebound follow up (Day 21); **(4) Rebound Heart Rate**: Change in heart rate from end of the target drug dose (Day 19) to rebound follow up (Day 21). **(5) Rebound Respiration**: Change in respiratory rate from end of the target drug dose (Day 19) to rebound follow up (Day 21); **(6) Long-term Glycemic Control**: Change in HbA1c and fructosamine levels from Day 2 to end of the target drug dose (Day 19).

### Study setting and recruitment

Patients were recruited from the Caron Treatment Center (CaronTC) in Wernersville, PA, USA. All patients were pre-screened at admission to determine potential eligibility. OUD patients who planned to remain in residential treatment for a minimum of 4 weeks at CaronTC were identified. All patients who met prescreen inclusion/exclusion criteria were approached for potential participation. The study was approved by the Penn State Hershey Medical Center IRB, and all participants signed IRB-approved consent forms after a full explanation of procedures, and prior to their engagement in any study procedures. If the patient agreed to participate, a study team member thoroughly reviewed the consent packet and answered any questions the participant may have had prior to the participant signing the consent forms. At the time of consent, they were enrolled in the study procedure which took 21 days to complete. Study enrollment did not interfere with or delay the administration of standard OUD therapies, e.g., behavioral treatments and/or buprenorphine/naloxone.

### Inclusion/exclusion criteria

Inclusion criteria included: (1) Age 18 to 75 years; (2) Diagnosed with an OUD, seeking treatment at CaronTC, and planning on being enrolled in a residential treatment plan for a minimum of 4 weeks; (3) Women of childbearing potential must consent to use a medically accepted method of birth control or to abstain from sexual intercourse while in the study; (4) Able and willing to provide informed consent prior to any study-related activities; (5) Able to read and communicate in English sufficiently to complete all study requirements. Exclusion criteria included: (1) Age < 18 or > 75 years; (2) Women who were pregnant, planning pregnancy, breastfeeding, or unwilling to use adequate contraceptive measures; (3) History of angioedema, serious hypersensitivity reaction, or anaphylactic reaction to liraglutide or another GLP-1RA; (4) Personal or family history of medullary thyroid carcinoma or patients with multiple endocrine neoplasia syndrome type 2 or thyroid nodule; (5) Type I diabetes or history of diabetic ketoacidosis; (6) Type II diabetes mellitus; (7) Hypoglycemia on intake visit (blood glucose < 70 mg/dL); (8) End-stage renal failure on dialysis or a glomerular filtration rate (GFR) < 30mL/min per 1.73 square meters, or previous renal transplant; (9) Severe hepatic impairment (AST or ALT levels > 3 times upper limit of normal range) or previous liver transplant; (10) Current or past diagnosis of pancreatitis, gastroparesis, or other severe gastrointestinal disease; 11) Current or past diagnosis of gallbladder disease or gallstones; 12) Serious cardiovascular disease within the past 6 months (e.g., uncontrolled hypertension, heart failure, significant cardiac arrhythmias, myocardial infarction, presence of angina pectoris, symptomatic coronary artery disease, deep vein thrombosis, pulmonary embolism, second- or third-degree heart block, mitral valve or aortic stenosis, hypertrophic cardiomyopathy, stroke); 13) Severe co-occurring psychiatric disorder (e.g., bipolar disorder, psychotic disorder, schizophrenia) that would, in the opinion of the study physician or Principle Investigator, interfere with participating in the study, such as the patient needing a higher level of care and/or requiring transfer out of Caron’s treatment facility; 14) Suicidal ideation within the past 1 month, or history of suicide attempts within the past 1 year, unless participation is cleared by clinician assessment; 15) Treatment with any investigational drug in the month preceding the study; 16) Previous randomization for participation in this trial; 17) Abnormal physical exam findings, vital signs (blood pressure, heart rate, respiratory rate, body temperature), EKG measurements, or safety lab values that were deemed clinically significant by the study physician.

### Trial design/procedure

#### Intervention allocation and blinding

Liraglutide 6 mg/ml (Saxenda®, Novo Nordisk) or placebo was provided as a solution in a pre-filled, multi-dose pen that delivered doses of 0.6 mg, 1.2 mg, 1.8 mg, 2.4 mg, or 3.0 mg of liraglutide as a subcutaneous injection. Study drug and placebo were supplied by Novo Nordisk as part of an approved investigator-initiated study (ISS-001291). To use Saxenda® as a blinded comparator in the clinical trial, study drugs were blinded to participants and investigators (double blind) through the distribution of identical injection pens. The solution color (clear), delivery mechanism (injection pen), and placement of injections (abdomen) were identical between the treatment groups. Injection pens were ordered from Novo Nordisk and received in boxes by an off-site pharmacy that supplied medications to CaronTC. Study drug packs were blinded by the pharmacy and delivered to CaronTC with a de-identified label and specific Dispensing Unit Number (DUN).

Laboratory staff analyzing the blood samples were offsite and unaware of the condition and the treatment of the participants; samples were identified only by study number. The results of the laboratory measurements were not shared with the treating physicians, and did not impact patient care except in an emergent situation.

#### Randomization

Following consent, participants were randomized to either active study drug or placebo conditions. Randomization was performed using a random number generator via the PLAN procedure within SAS software, version 9.4 (SAS Institute Inc., Cary, NC). A randomization list was created using variable-size, random permuted blocks to ensure that the number of participants in each arm was balanced after each set of B randomized participants, where B is the block size. The study biostatistician chose the block sizes without revealing it to any investigators or study personnel who were collecting and reviewing outcome data. The randomization sequence was uploaded to a secure REDCap database. The distributing pharmacy was given access to this REDCap database to provide the blinded, randomized labelling prior to shipping the study drugs to the study site.

In the case of a medical emergency, when knowledge of the participant’s treatment assignment might influence the participant’s clinical care, the treating physician was able to contact the study pharmacy to access the participant’s treatment assignment. Research team members documented the reasons for unblinding in the participant’s source documentation. Study team members did not share the information with personnel involved with the analysis and conduct of the study.

#### Participant timeline

To facilitate recruitment for a 21-day protocol within a typical 28-day residential stay, participants meeting prescreening criteria were identified through the electronic medical record (EMR) and approached within the first 3–5 days of their treatment initiation. Participants were considered out of withdrawal and able to begin the study when the treating physicians at Caron transferred the participant from the withdrawal unit at Caron to the program treatment unit. All participants provided written informed consent after an explanation of study procedures, following the protocol endorsed by the University’s Institutional Review Board.

Following consent on Study Day 1, participants completed physiological and psychiatric screening measures relevant to study inclusion/exclusion criteria. Physiological measures included a 12-lead EKG and baseline assessment of cardiorespiratory function. Comprehensive metabolic panel (CMP) and complete blood count (CBC) were obtained from the EMR. Psychiatric screening and assessments included the Mini-International Neuropsychiatric Interview (M.I.N.I. 7.0.2) [59], Columbia Suicide Severity Rating Scale (C-SSRS; [60]), Form 90-DI [61], Hamilton Depression Rating Scale (HAM-D; [62, 63]), and State-Trait Anxiety Inventory (STAI-Y1; [64]).

Participants that passed the screening process were randomized to either the liraglutide or placebo treatment arm of the study using an equal allocation ratio (1:1) permuted-block randomization, as described above. Following randomization, participants were fitted with a wristband actigraph to collect physiological measures of stress response, activity, and sleep. Participants also rated their craving on a visual analogue scale (VAS) during a cued-response task while their prefrontal cortex was being monitored with fNIRS. Documentation of adverse events (AEs) and concomitant medications was also initiated on Day 1.

The study medication/placebo was administered and body weight recorded each day prior to breakfast on Study Days 2–19. Cardiorespiratory assessment was conducted on Days 2, 8, 14, and 19 (the first day of each dose, post-test day). EMA assessments were collected on Days 2–3, 6–9, 12–15, 18–19 (first and last 2 days of each dose). Stress response, sleep, and activity measurements were collected via actigraphy wristband on Study Days 2–19.

Days 20–21 served as a rebound period following discontinuation of the study medication. Body weight and EMA were collected both days, and a final cardiorespiratory assessment was conducted on Day 21. All participants then received an End of Study telephone call 30 days (+/- 5 days) after the discontinuation of study medication during which participants were asked to self-report their body weight and any adverse events they may have experienced. Table 1 demonstrates the schedule for the trial.

**Table 1:**
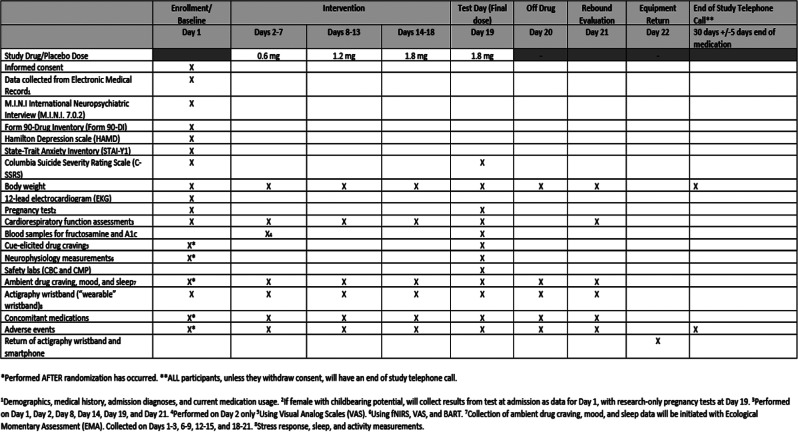
Schedule of study activities

### Measures

#### Screening and assessments

Day 1 screening and assessments included the following. The Form 90-DI was used to evaluate patterns of drug use and drug history and to document polysubstance use in the prior 3 months. Although opioid use was the primary inclusion criterion (i.e., OUD diagnosis) for the study, the use of additional substances was not exclusionary as polysubstance use is commonplace in substance use disorders. Psychiatric disorders were assessed using the MINI. The C-SSRS, was used to screen for past 1-month suicidal ideation, and past 1-year suicide attempts. Severity of depression was assessed using the HAM-D, and anxiety was assessed using the STAI-Y1.

A 12-Lead EKG (McKesson Lumeon series Electrocardiograph Eli 280), conducted by the study nurse and interpreted by the study physician, was used to assess cardiac function.

#### Cardiorespiratory assessment

Cardiorespiratory assessments were conducted using the AD Instruments Power Lab 8/35 data acquisition system with Human NIBP Nano Interface and Bio Amp modules. LabChart 8 Pro (v8.1.13) software collected respiratory rate via pneumography belt, heart electrical activity via EKG, and blood pressure via finger cuff (photoplethysmography). Arm blood pressure was measured using an Omron BP7100 blood pressure monitor, and blood oxygenation using a Smiths Medical BCI 3301 pulse oximeter.

#### Actigraphy wristband

Stress response, sleep, and activity measurements were continuously assessed from Day 1 through Day 21 using a fitness activity tracker (Garman Vivosmart 4). Fitness and health monitoring tools included wrist-based heart rate, all-day stress tracking, VO_2_ max (maximal oxygen consumption), REM sleep, and blood oxygen saturation (pulse O_2_). The study coordinator instructed each participant on use of the wristband.

#### Ecological momentary assessment (EMA)

Ambient drug craving, mood, and sleep data were collected using the Wear-IT framework [65]. Frequency and strength of craving were assessed on 0-100 point Likert scales. Three items from the Desire for Drugs scale (adapted from the Desire to Drink scale, [66]) were assessed on 0–4 scales to remain consistent with the prior literature. These items included: “The idea of using drugs has intruded upon my thoughts”; “I have thought about how satisfying drugs can be”; and “I have missed the feeling drugs can give me”.

#### Visual analogue scale (VAS)

Immediately prior, and following, administration of the cue reactivity task, participants were asked three items, on a 0-100 scale, to assess cue-induced craving in response to the drug cues presented in the task. The items included: “How much do you want to use right now?”, “How much do you want to avoid using right now?”, and “How much control do you feel you have over using right now?”.

#### Functional near infrared spectroscopy (fNIRS)

Participants completed a cue reactivity task and the Balloon Analogue Risk Task (BART) while the prefrontal cortex was monitored with fNIRS. FNIRS is a neuroimaging technology that uses near infrared light to monitor changes in the concentration of oxygenated and deoxygenated hemoglobin in the brain, analogous to fMRI [67–71] but with wearable and potentially mobile sensors consistent for monitoring in increasing ecologically valid settings [72, 73]. During the cue reactivity task, participants passively viewed six types of imagery, including: positive social interactions (e.g., a happy family at the park), highly palatable food (e.g., a hot fudge sundae), non-erotic images of emotional intimacy (e.g., a romantic couple holding hands), drug-related imagery (e.g., opioid packaging or pills), drug control imagery (e.g., objects similar in shape and size to pills, but not drug-related), and emotionally neutral images (e.g., a chair or lamp). The BART is a decision-making task designed to assess risk-taking behaviour. Participants were able to earn virtual rewards with each inflation of a digital balloon. Increased responding accumulated reward but also increased the chance the balloon would “pop”, resulting in loss of all gained reward. All participants played an active version of the BART where they were free to respond, as well as a passive version in which the participant had to watch the computer complete the task for them. Active and passive versions were counter-balance across participants for order of presentation. All imagery and tasks were presented using E-Prime 3 software (version 3.0.3.82, Psychology Software Tools Inc.).

FNIRS data were recorded using a 4 × 10 (4 light-emitting diodes and 10 photodetectors) optode set with 16 channels and a continuous wave system (fNIR Devices, Model 2000 S). The fNIRS sensor was fitted by aligning the bottom row of optodes with the International 10–20 sites F7, FP1, FP2, and F8 line which was situated over the rostral and ventrolateral prefrontal cortex. Raw light intensity data from the 16 optodes and two wavelengths were low-pass filtered using a finite impulse response, and was linear phase filtered with order 100 and a cut-off frequency of 0.1 Hz to reduce high frequency noise, respiration, and the cardiac cycle effect. A sliding motion artifact rejection (SMAR, [74]) statistical filter was employed for motion artifact rejection, and data were inspected for potential saturation effects and motion artifact contamination by an evaluator blind to participant status. The data from each block was extracted and hemodynamic changes for each optode was calculated using the Modified Beer Lambert Law. The final output used for analyses will be mean oxygenated hemoglobin (HbO2) at each of the 16 optodes for each stimulus type minus neutral images.

#### Study treatment dose regimen

Each participant received increasing doses of liraglutide/placebo over the course of 18 days. Study medication started at a low dose, and then escalated every 6 days, in an effort to minimize side effects, in particular gastrointestinal distress. For Days 2–7, participants received 0.6 mg daily; on Days 8–13, 1.2 mg daily, and on Days 14–19, 1.8 mg daily. Each time a new dose was started, a cardiorespiratory assessment was conducted and AEs and concomitant medications continued to be documented daily. Participants were not able to deviate, halt, or modify the planned dose escalation, per protocol regulation; in the event of prohibitive AEs, participants were withdrawn from the study.

Adverse events were defined as “any untoward medical occurrence associated with the use of the drug, whether or not considered drug related” and were monitored continuously throughout the study. AEs were initially identified daily by the study nurse and study coordinators, and were assessed in conjunction with the study physician and in discussion with the PI. Briefly, the study nurse asked the participants daily, during the study medication visit, whether they had experienced any adverse events; study coordinators also inquired about adverse events during visits with the participants. Additionally, study coordinators reviewed the participants’ medical record daily for unreported adverse events. All adverse events (serious or non-serious) and abnormal test findings observed or reported to study team, which were believed to be associated with the study drug, were followed until the event (or its sequelae) or the abnormal test finding resolved or stabilized at a level acceptable to the investigator. The investigator reported to The Pennsylvania State University Institutional Review Board (IRB) any observed or reported harm (adverse event) experienced by a participant which, in the opinion of the investigator, was determined to be [1] unexpected and [75] possibly or probably related to the research procedures.

### Data management and monitoring

#### Early withdrawal of subjects and safety reporting

Participants will be withdrawn if they experience: (1) Systolic blood pressure changes > 40 mmHg, to levels < 90 mmHg or > 200 mmHg, or clinical symptoms of hypertension (e.g., headache, dizziness, blurred vision, nausea, edema) or hypotension (e.g., dizziness, nausea, fainting); (2) Heart rate reached levels < 40 beats/min or > 120 beats/min; (3) Respiratory rate changes by > 5 breaths/minute or reached levels < 10 breaths/minute or > 24 breaths/minute; (4) The investigators will also exercise discretion to end an individual’s study participation if they engage in behavior that could jeopardize their own health and well-being or that of others; (5) Participants that develop any new conditions described in the exclusion criteria during the treatment phase of the clinical trial will be removed from the study; (6) Participants will be withdrawn from the study if any study participant withdraws consent for any reason; the primary reason for the subjects’ withdrawal from the study will be recorded.

#### Data and safety monitoring plan

The data safety monitoring board (DSMB) was responsible for safeguarding the interests of participants in this trial. This responsibility was exercised by providing recommendations for continuation or early termination of the trial, based on assessment of safety. The DSMB also formulated recommendations related to the selection, recruitment or retention of participants, their management and adherence to protocol specified interventions, and the procedures for data management and quality control. The DSMB was advisory to the PI, to the medical monitor, and to their co-investigators. The PI was responsible for promptly reviewing and implementing DSMB recommendations. The DSMB consisted of at least 3 independent experts that had no conflicts of interest.

#### Clinical trial monitoring plan

The goal of the Clinical Trial Monitoring Plan was to ensure clinical trial activities followed the approved study protocol, met institutional policies and requirements, and adhered to good clinical practice guidelines. REDCap projects utilized pre-defined user role assignments to control and limit access to the data within the project. The audit file listing data viewed, entered, modified and changed was listed within the logging module.

The randomization was performed in a separate REDCap project and only the pharmacy had access to that project. Data entry began on the day the informed consent was executed and continued through days 1–19 of the treatment period, days 20–21, and the End of Study Telephone call (30 days post treatment). The REDCap project utilized double data entry practice for quality control. The source documents for the study included paper case report forms, electronic data capture from devices, the electronic medical record, laboratory reports, and EKG results. Participant information and paper records were stored in a secured filing cabinet in a locked room on site, with limited access. All reports, data collection, process, and administrative forms were identified by a coded ID number only to maintain participant confidentiality. All records that contained names or other personal identifiers, such as informed consent forms, were stored separately from study records identified by code number. Additional protocol training was required by all team members following each protocol modification. Regulatory documents were reviewed every six months. Periodic onsite monitoring visits and remote visits to the Pharmacy were conducted following the 1st, 5th, 15th and 30th randomized participants. A final close out visit was conducted.

### Statistical analysis

The goal of this study is to provide pilot data for a larger clinical trial. This study aimed to enroll 40 OUD patients in residential treatment. To account for an estimated 20% attrition rate, we planned to randomize a total of 20*2/0.8 = 50 patients in the study. A sample size of *n* = 20 per group would give 80% power to detect an effect size of 0.91 based on a two-sided two-sample t-test with a significance level of alpha = 0.05. To preserve statistical power, the sample was not balanced with regard to MOUD and non-MOUD participants.

Although recruitment and study protocol are complete, detailed analyses on endpoints and specific variables are currently ongoing and will be presented in future publications. The primary aim is to test whether liraglutide reduces craving in patients recovering from OUD in residential treatment. Analysis of covariance (ANCOVA) will be used to compare liraglutide versus placebo treatment groups controlling for baseline values of craving. Linear mixed effects models for repeated measurements will also be used to evaluate the difference in changes in these primary outcomes between treatment groups. The baseline and post-treatment outcomes are treated as dependent variables in the mixed model. Subjects with missing values can be incorporated in the mixed model analysis when data are missing at random. The moderating effect will be assessed by adding the interaction term between moderator and treatment group in the models. SAS (version 9.4) and R (version 3.2.5) software will be used for analyses with a significance level of 0.05.

Summary statistics including means, medians, ranges, and standard deviations will be computed for each continuous variable and frequencies with percentages for categorical variables. Per intention-to-treat principles, all collected data will be included in statistical analyses wherever possible, regardless of outlying values. Primary outcomes to assess efficacy are: [1] cue-elicited drug craving rated on a 100-pt VAS; and [75] ambient drug craving, derived from EMA of daily craving throughout the study period. Data analyses for cue-elicited craving will consist of a 2 × 2 ANOVA with medication status (pre- vs. post-medication) as a within-group variable and medication type (liraglutide vs. placebo) as a between-group variable. Secondary analyses of self-reported craving measures will evaluate the role of MOUD, specifically BUP/NA, in these results.

#### Interim power analysis

Although an interim data analysis was initially planned, no interim analyses were conducted during the study - specifically to avoid any reduction in power, as complications related to the pandemic significantly affected participant recruitment. In its place, an interim power analysis was conducted to determine, based on the data collected at that time, the effect size needed to observe a significant drug effect. In particular, we examined the precision of the estimates in order to determine power. We currently have a manuscript in preparation detailing the theoretical underpinnings of this approach [76]; this concept is similar to a currently published model [77]. This analysis did not bias type 1 error rates and the study team used this information to decide that there was sufficient power to detect a medium sized effect in the final analyses, and to forego any interim analyses.

## Discussion

This manuscript describes a protocol for a randomized, double-blind, placebo-controlled pilot study to evaluate the safety and efficacy of the GLP-1RA, liraglutide, as a treatment for individuals with OUD. To date, GLP-1RAs have not been evaluated clinically in OUD, despite a growing body of preclinical data supporting efficacy in such a role. The current protocol was the first to examine the clinical relationship between liraglutide and craving for opioids in a residential treatment setting. Because it is a multidimensional phenomenon with subjective, physiological, neurochemical, and behavioral correlates, craving was assessed in three ways; in-the-moment ambient craving was assessed 4 times daily throughout the study using programmed smartphones (EMA), cue-induced craving was assessed in response to opioid-related visual cues in a cue-reactivity paradigm before and after the intervention, and objective, neurophysiological responses to the opioid cues were assessed using fNIRS neuroimaging during these same cue-reactivity tasks. In addition to efficacy, the current protocol is also the first to explore the safety of extending liraglutide into a sample population undergoing treatment for OUD.

### Trial-related events

A number of trial-related events, both anticipated and unanticipated, were identified prior to, and during, recruitment for the pilot study. First, an optional component was added to the protocol to assess whether liraglutide would be effective in the treatment of the dysregulated sleep often observed in OUD patients, as funded by a grant supplement (UG3 DA050325-02S1). Sleep disturbances are an extremely common and poorly treated problem in recently detoxified SUDs, in particular OUD patients. Previous studies of OUDs report a point prevalence in sleep disturbances of 60 to 90% of OUD subjects who are identified as post-detoxification and in the early recovery period [78–81], and in persistent abstinence [78, 82–84]. As GLP-1RAs can decrease orexin neurotransmission [85], and such a decrease can dampen arousal and lead to improved sleep disturbances, we proposed that liraglutide may not only decrease craving, but improve sleep by acting on the arousal pathways as well. To that end, an optional component was created to administer a level 2 polysomnography (PSG) recording and the Insomnia Severity Index (ISI) scale on Day 1 (pre-treatment) and Day 19 (post-treatment) of the protocol; PSG and ISI are objective and subjective standards, respectively, for assessing sleep and insomnia symptoms and therapeutic effects in the field [86–89].

Second, we initially proposed to use a commercially available continuous glucose monitoring (CGM) device to track interstitial glucose levels in real time, 24 h/day, to address the concern of using a medication for glycemic control in a non-diabetic population (i.e., producing hypoglycemic events). However, use of CGM was discontinued after a number of participants experienced numerous unsubstantiated and disruptive urgent low alerts (glucose < 54 mg/dl) in the beginning of the trial, prompting their early withdrawal due to frequent finger sticks required for verification of blood glucose levels; blood glucose levels immediately following each urgent low alert were verified using a glucometer and all readings were > 75 mg/dl, well above urgent low levels; participants did not endorse any symptoms of hypoglycemia. The cause of these alert anomalies has yet to be established, and an interesting basis for a follow-up study would be to determine whether innate or interacting factors specific to an OUD population were responsible, as these false alerts have not been observed in general or diabetic populations. However, for the current protocol, CGM use was discontinued for a number of reasons. First, the limited, preliminary safety data that was collected in the current protocol before CGM discontinuation, raised no concerns regarding hypoglycemia. Second, to further support the reasonable discontinuation of CGM in the trial, concerns regarding potential hypoglycemic events in a non-diabetic population are mitigated by the current safety record of GLP-1RAs in the obesity population [90]. Finally, as a matter of practicality, the fingersticks involved in frequent verification of blood glucose levels drove some participants out of the study (i.e., prompted early withdrawal).

### Key strengths and limitations

The fact that the current protocol was designed as a pilot study, and set in a residential treatment facility, presented some limitations, but also some strengths as well. The current stay for residents at CaronTC is approximately 28 days; given that initial withdraw and stabilization can take a week, the study had to be conducted within a 3-week timeframe. This meant modification of the standard liraglutide dosing regimen. Whereas the standard regimen involved seven days of each escalating dose (0.6 mg, 1.2 mg, 1.8 mg, 2.4 mg, and 3.0 mg; total 35 days), the current protocol was limited to 6 days of the first three doses each (0.6 mg, 1.2 mg, 1.8 mg; total 18 days), to align with the timeframe of their stay. However, the 1.8 mg dose of liraglutide is an effective and standard treatment dose for Type 2 diabetes mellitus and, while the dosing regimen in the current protocol did not include the higher doses (i.e., 2.4 mg, 3.0 mg), preclinical data suggests that lower liraglutide doses may be effective in animal models of opioid seeking and taking (38, unpublished data). Additional studies will be needed to evaluate the necessity and efficacy of higher doses.

As the safety of liraglutide in an OUD population was an endpoint of the current protocol, a major strength of conducting this study in a residential treatment setting was the ability to monitor participants and to have medical staff available 24 h/day to address potential medical concerns. If liraglutide is determined to be safe in this population, with negligible or minimal adverse events, then the current protocol can provide a template for a follow-up project in a larger, Phase 2 clinical trial.

Future study designs would also benefit from the collection of plasma samples for pharmacokinetic (PK) analyses during the rebound period, although PK analyses were outside the scope of the current protocol. The aim of the rebound period in the current study was to assess whether immediate cessation of the medication was associated with any safety concerns (there were no such concerns observed). The current pharmacokinetics literature for liraglutide suggests that plasma levels were not high during the timeframe that corresponded to the rebound period. The half-life for liraglutide is 13 h in humans, with an accumulation index of 1.5 [91]. As the final dose of liraglutide was administered in the morning on Day 19, ~ 75% of the plasma levels should have been cleared by the end of the first rebound day (Day 20). That said, PK analyses would be beneficial and would provide critical information and a more empirical assessment of drug levels for future study designs.

Finally, a known, but unavoidable, limitation to the current study design is the use of pre- and post-treatment assessment of certain measures (e.g., the cue-induced craving endpoint, VAS, glycemic control, etc.). Patients in residential treatment for SUD may not be able to complete treatment and often will leave early, against medical advice. In the current study, this frequently resulted in a problematic research environment with a high number of participant withdrawals leading to missed data collection on post-treatment measurements. Balancing participant burden and analytical power for collecting intensive longitudinal data was challenging in this setting, as certain measures were prioritized for daily data collection in an environment prone to high dropout rates, while other measures collected pre- and post-treatment were more limited to strictly paired assessment. The reliance on pre- and post-measures for certain variables does limit analyses due to the higher than expected withdrawal rate. However, as the current study is preliminary, the strategy for future analyses will be to conduct pre-post analyses only on complete cases (i.e., where both pre- and post-intervention data are available for each participant) for those variables. This will limit analyses to a smaller, but still adequate, number for preliminary analysis and will not necessitate carry-forward rules. Fortunately, this issue is somewhat mitigated for our repeated measures outcomes, including the primary endpoint of ambient craving as assessed via EMA. The collection of these measures was more frequent (4x daily) and continuous across the trial (compared with pre/post measurement), allowing mixed-effects analyses that account for missingness under the assumption of missingness at random.

### Anticipated impact

Although preclinical models suggest that the use of a GLP-1RA significantly reduces heroin and fentanyl seeking and taking in rats, no GLP-1RA is currently approved to treat OUD in humans. Completion of the current protocol could provide the initial clinical data with which to evaluate the potential use of GLP-1RAs as a medication for OUD in larger, outpatient studies. The results of this study could provide a first step in assisting physicians, third party payers, and health care agencies in including GLP-1RAs as a treatment option for opioid use disorder. Improved understanding of innovative, non-opioid based treatments for OUD has the potential to save and improve lives by expanding the number of diverse, safe, and effective treatments for OUD.

## Data Availability

Upon reasonable request, which should be made to the corresponding author, study data or materials may be made available.

## Abbreviations

GLP-1RA: Glucagon-like peptide 1 receptor agonist
SUD: Substance use disorder
OUD: Opioid use disorder
CGM: Continuous glucose monitoring
EKG: Electrocardiogram
PSG: Polysomnography
ISI: Insomnia Severity Index
EMA: Ecological momentary assessment
fNIRS: Functional near infrared spectroscopy
VAS: Visual analogue scale
BART: Balloon analogue risk task
ANOVA: Analysis of variance
ANCOVA: Analysis of covariance
BUP/NA: Buprenorphine/naloxone
MOUD: Medication for opioid use disorder
DSMB: Data and safety monitoring board
PI: Principle investigator
FDA: Food and drug administration
BMI: Body mass index
EMR: Electronic medical record
